# The effect of palonosetron hydrochloride in the prevention of chemotherapy-induced moderate and severe nausea and vomiting

**DOI:** 10.3892/etm.2013.996

**Published:** 2013-03-08

**Authors:** JIAN HUANG, XIAO-JIA WANG, DING YU, YE-NING JIN, LEI-ZHEN ZHEN, NONG XU, WEI LIU, YONG-CHUAN DENG, SHI-XIU WU, JIA HE

**Affiliations:** 1 Zhejiang Cancer Hospital, Hangzhou 310022;; 2 Hubei Cancer Hospital, Wuhan 430079;; 3 Ruijin Hospital, Shanghai Jiao Tong University School of Medicine, Shanghai 200025;; 4 Xinhua Hospital, Shanghai Jiao Tong University School of Medicine, Shanghai 200092;; 5 The First Affiliated Hospital of College of Medicine, Zhejiang University, Hangzhou 310009;; 6 Hebei Cancer Hospital, Shijiazhuang 050011;; 7 The Second Affiliated Hospital of College of Medicine, Zhejiang University, Hangzhou 310008;; 8 The First Affiliated Hospital of Wenzhou Medical College, Hangzhou 325000;; 9 Health Statistics Teaching and Research Section, The Second Army Medical University, Shanghai 200433, P.R. China

**Keywords:** palonosetron, granisetron, chemotherapy, nausea, vomiting

## Abstract

The current study aimed to evaluate the efficacy and safety of palonosetron hydrochloride injection for preventing chemotherapy-induced moderate and severe nausea and vomiting. A multi-centered, randomly stratified, double-blind, double-dummy, parallel-group and positive-controlled trial was performed. A total of 240 patients who underwent chemotherapy treatment which induced moderate or severe vomiting were divided into the experimental and control groups. Half an hour before chemotherapy, the experimental group received a 0.25-mg palonosetron hydrochloride injection, whereas the control group received a 3-mg granisetron injection. The acute vomiting complete remission rate (CRR) of the experimental group was not significantly different compared with that of the control group (P=0.35). The delayed vomiting CRR of the experimental group was significantly higher compared with that of the control group (P=0.002). No difference in full course vomiting CRR, vomiting control time, treatment failure time or acute nausea CRR was identified between the two groups. No significant differences in adverse events were observed between the experimental group and the control group. No significant differences in adverse reactions occurred between the experimental group and the control group (12.50%). Palonosetron hydrochloride injection had a better effect on delayed vomiting CRR than granisetron hydrochloride injection. The two injections exhibited similar effects on acute vomiting CRR, full course vomiting CRR, vomiting control time, treatment failure time (days), acute nausea CRR and adverse events.

## Introduction

Chemotherapy is one of the main treatment methods for numerous types of cancer. However, it is capable of inducing the release of 5-HT_3_ from enterochromaffin cells, which interacts with its receptors to cause vagal afferent nervous excitation, leading to the vomiting reflex. Nausea and vomiting are the two most common adverse reactions in cancer patients who receive chemotherapy. Inadequate control of these reactions often leads to a series of associated complications, which in turn may affect treatment outcome and compliance. Significant progress has been made in the prevention and treatment of chemotherapy-induced nausea and vomiting due to the application of 5-HT_3_ receptor antagonists. Commonly used first-generation 5-HT_3_ receptor antagonists include ondansetron and granisetron. Although these drugs are able to achieve a complete control (CC) rate of 50–70% for acute vomiting (1–5), they are not as effective in delayed vomiting*,* even in scenarios with repeated or combined medication plans (2). Therefore, the development of a more effective drug is urgently required.

Palonosetron hydrochloride injection belongs to the highly selective second generation of 5-HT_3_ receptor antagonists. It was first developed by the the Helsin Healthcare S.A. Company (Lugano, Switzerland) and appeared on the market in the USA in July 2003 with the trade name Aloxi™ (6). However, whether this drug has a good curative effect or is safe for use among the Chinese population is unclear.

Therefore, a multi-centered clinical trial was conducted, with the support of the State Food and Drug Administration of China (no. 2007L00939) between September 2009 and September 2010. The drug involved in this study was generic palonosetron hydrochloride injection (a new drug produced by Zhejiang Puluo Kangyu Natural Medicine Co., Ltd., Jinhua, China).

## Subjects and methods

### Subjects

Selection criteria for the subjects were as follows: i) They were histologically or cytologically diagnosed with malignant tumors (without tumor type restriction) and were required to receive chemotherapy; ii) no restrictions were imposed on their chemotherapy plan, which involved the application of chemotherapeutic drugs known to induce moderate or severe vomiting [the severity of chemotherapy-induced nausea was defined according to the NCCN Guidelines^®^ for Antiemesis (8)]; iii) the patients did not receive any additional treatments, particularly chemotherapeutic drugs, from days 2–7 in a cycle of chemotherapy or antiemetics, tranquilizers, psychostimulants, antihistamines or hormones from days 1–7; iv) their age ranged from 18 to 75 years for either gender and their Karnofsky performance status scores were ≥60; v) their life expectancy was >3 months; vi) their bone marrow was able to produce blood sufficiently; vii) they had normal liver and renal functions; viii) their electrocardiograms were basically normal, which satisfied chemotherapeutic eligibility; ix) they had recovered from toxic reactions (with the exception of baldness and nail changes) induced by previous treatment at least three weeks before the last time of radiotherapy and chemotherapy; and x) they had signed an informed consent form. The study was approved by the ethics committee of Zhejiang Cancer Hospital, Hangzhou, China.

### Study design

Since granisetron hydrochloride injection, which is currently administered in clinics, is apt to reduce nausea and vomiting, this study adopted a multi-centered, randomly stratified, double-blind, double-dummy, parallel-group and positive-controlled trial method. Based on stratification factors, including the severity of chemotherapy-induced nausea, gender and whether or not this was the first time the patient was receiving chemotherapy, the palonosetron hydrochloride injection (experimental) and granisetron hydrochloride injection (control) groups were created, with 120 patients allocated to each group. Eight centers were included in the study through competition and the central randomization system was applied.

### Investigational drug

Palonosetron hydrochloride injection was provided by Zhejiang Puluo Kangyu Natural Medicine Co., Ltd. (batch no. 090504), whereas granisetron hydrochloride injection was provided by Ningbo Teampharm Co., Ltd. (batch no. 090301, Ningbo, China). A double-blind and double-dummy method was adopted. The experimental group were administered palonosetron hydrochloride injection (0.25 mg) plus a granisetron hydrochloride injection simulated agent, whereas the control group were administered granisetron hydrochloride injection (3 mg) plus a palonosetron hydrochloride injection stimulating agent. Randomization was performed using SAS software, in accordance with the stratification factors. An emergency letter for unblinding was prepared for each patient. The drugs were diluted with physiological saline to 40 ml and administered to the patients half an hour before chemotherapy. The time taken to administer intravenous injection was >5 min.

### Evaulation of curative effect

The curative effect for vomiting was evaluated according to the following criteria (7,8): i) Complete remission (CR), 0 times/24 h; ii) partial remission (PR), 1 time/24 h; iii) mild remission (MR), 2–5 times/24 h; and iv) failure (F), >5 times/24 h. The remission rates were calculated as follows: CR rate = number of vomiting-free cases/total number of cases; PR rate = number of PR cases/total number of cases; and the effective rate = number of CR+PR+MR cases/ total number of cases. The three rate indices were calculated according to three observation intervals of acute (0–24 h), delayed (24–120 h) and full course (0–120 h) vomiting, respectively.

The effect on nausea was evaluated according to the following criteria: i) CC, normal and nausea-free; and ii) partial control (PC), poor appetite with no changes in food habits (mild nausea) or decreased in food intake, no marked weight loss, dehydration or malnutrition and infusion time of ≤24 h (moderate nausea). The control rates were calculated as follows: CC rate = number of CC cases/total number of cases and PC rate = number of CC+PC cases/total number of cases.

### Safety indices

The patients were observed every day during the trial and all adverse events were carefully recorded. Examination of vital signs, electrocardiograms, blood routine, urine routine, liver function, renal function and electrolyte levels were performed prior to and after treatment for safety. Adverse events were evaluated according to the National Cancer Institute CTCAE v3.0 criteria (9,10).

### Statistical analysis

Biostatistical analysis was performed by an independent third party (the Health Statistics Teaching and Research Section of The Second Army Medical University, Shanghai, China) using SAS 10.0 software. All rejected and suspended cases were statistically described. Demographic data and other basic indices were compared between the two groups using the χ^2^ test (or Fisher’s exact probability test), Cochran-Mantel-Haenszel (CMH) test, Student’s t-test and variance analysis or nonparametric statistical analysis methods. Test methods, including logistic regression analysis, the CMH test and covariance analysis, were carried out to the main curative indices for non-inferiority analysis and the non-inferiority threshold was determined to be 15% (Δ=15%). Adverse events in the two groups were statistically described and compared using the χ^2^ test or the Fisher’s exact probability test. Comparisons of vital signs, laboratory examination and electrocardiogram results were based upon the statistical description and analysis of changes observed prior to and after treatment. The mean values and incidence rates before and after drug administration were compared when necessary. α=0.05 was used for all hypothesis tests.

## Results

### General data

A total of 240 patients were involved in this study and they were evenly divided into the experimental and control groups (n=120). A total of 117 cases in the experimental group and 119 in the control group were enrolled in the full analysis set (FAS), 114 cases in the experimental group and 116 in the control group were enrolled in the per-protocol population set and 118 cases in the experimental group and 120 in the control group were enrolled in the safety analysis data set. The number of suspended cases in the experimental and control groups were 1 and 0 and the number of rejected cases were 7 and 4, respectively; no significant differences were identified. The demographic and baseline characteristics and vital signs of the two groups were similar and no significant differences were identified (Tables I and II).

### Curative effect

No significant difference was identified between the acute vomiting CR rates of the experimental and control groups (49.12 vs. 42.24%, respectively; P=0.350; 95% CI, -5.96-19.73; Table III). The non-inferiority test showed that palonosetron hydrochloride injection was not inferior to granisetron hydrochloride injection (P<0.001).

A significant difference was identified between the delayed vomiting CR rates of the two groups (51.75 vs. 31.03%; P=0.002; 95% CI, 8.27–33.17; Table IV). The non-inferiority test showed that palonosetron hydrochloride injection was not inferior to granisetron hydrochloride injection (P<0.001).

No significant difference was identified between the full course vomiting CR rates of the two groups (37.72 vs. 27.59%; P=0.121; 95% CI, -1.92-22.19; Table V). No significant difference was identified between the vomiting control times (7.62±11.68 vs. 9.72±16.01; P=0.573; Table VI), treatment failure times (days; 1.41±1.01 vs. 1.36±0.70; P=0.712; Table VII) or the acute vomiting CR rates (32.46 vs. 27.59%; P=0.136; Table VIII) of the two groups.

### Safety

Adverse events in this study included any diseases, newly-emerged symptoms, abnormal vital signs or laboratory results and the aggravation of original symptoms or vital sign abnormalities occurring during the clinical drug trial, regardless of whether they were associated with the investigational drugs. Severe adverse events included death, threat to life, permanent or definite disabilities or handicaps, hospitalization or the extension of hospitalization length, congenital disabilities or birth defects, drug overdose and any other severe medical emergencies. Significant adverse events include those listed in adverse events and evident hematological or other laboratory test result abnormalities during drug administration, which may only be cured through targeted treatment.

In the experimental and control groups, 68 (57.63%) and 81 cases (67.50%), respectively, experienced adverse events, 51 (43.22%) and 51 cases (42.50%), respectively, experienced significant adverse events and 0 (0.00%) and 1 case (0.83%), respectively, experienced severe adverse events; no significant differences were identified between the two groups. Adverse reactions in the experimental and control groups were experienced in 17 (14.41%) and 15 cases (12.50%), respectively, and no significant difference was detected between the two groups (Table IX).

Adverse events which occurred at an incidence rate of >10% included leucopenia (11.02% in the experimental group and 22.50% in the control group) and myelosuppression (8.47% in the experimental group and 10.83% in the control group). These events were correlated with the bone marrow functionality of patients, as opposed to with the investigational drugs.

Grade 4 myelosuppression was the one severe adverse event which occurred in the control group, however this was not correlated with the investigational drug and was eventually cured.

Adverse reactions which were associated with the investigational drugs included constipation, asthenia, diarrhea, dizziness and abdominal distention. No statistically significant differences were observed between the two groups. The incidence rates of these reactions in either group were not >10%.

## Discussion

The current study aimed to compare the efficacy and safety of palonosetron hydrochloride injection and granisetron hydrochloride injection in the prevention of chemotherapy-induced moderate and severe gastrointestinal reactions. It was a multi-centered, double-blind, double-dummy, parallel-group, positive-controlled clinical trial. Chinese patients were administered palonosetron hydrochloride injection (0.25 mg) or granisetron hydrochloride injection (3 mg) half an hour before chemotherapeutic drug administration, for observation of the efficacy and safety of palonosetron hydrochloride injection in preventing chemotherapy-induced nausea and vomiting.

Between 22 September 2009 and 7 September 2010, a total of 240 patients from eight trial centers were investigated. They were divided into the experimental and control groups through central randomization according to certain factors, including gender, whether this was the first time the patient had received chemotherapy and the severity of chemotherapy-induced nausea. The demographic and baseline characteristics of the two groups were similar.

The results in this study demonstrated that there was no significant difference between the acute vomiting CR rates, full course vomiting CR rates, vomiting control times, treatment failure times, acute nausea CR rates or adverse events of the two groups, however, palonosetron hydrochloride injection did exhibit a better control on delayed vomiting compared with palonosetron hydrochloride injection. These results were identical to those previously reported (11), indicating that this drug has a good effect and high safety amongst the Chinese population.

Palonosetron has a 10–100 times higher affinity for 5-HT_3_ receptors than other 5-HT_3_ receptor antagonists (the pKi of palonosetron was 10.4 and those of granisetron, tropisetron and ondansetron are 8.91, 8.81 and 8.39, respectively) (12) and has an elimination half-life of 40 h, which was markedly longer than that of other 5-HT_3_ receptor antagonists (ondansetron, tropisetron and granisetron have half-lives of 4, 7.3 and 8.9 h, respectively) (13–15). Therefore, palonosetron not only prevents chemotherapy-induced acute nausea and vomiting, but also has a positive preventative effect on delayed nausea and vomiting (16).

Since the single use of 5-HT_3_ receptor antagonists is not able to completely control chemotherapy-induced nausea and vomiting, the option of an increase in the 5-HT_3_ receptor antagonist dose or therapeutic combination with other drugs, for example glucocorticoids or H_2_ receptor antagonists, is often selected in clinical practice to enhance antiemetic effects (17–19).

Although antiemetic drugs with differing mechanisms of action, for example, the neurokinin-1 receptor blocker aprepitant (20), have already appeared on the worldwide market, 5-HT_3_ receptor antagonists remain the major drugs used for antiemesis.

In conclusion, compared with granisetron hydrochloride injection, palonosetron hydrochloride injection has a better effect on delayed vomiting among the Chinese population. The two injections have a similar effect on the acute vomiting CR rate, full course vomiting CR rate, vomiting control time, treatment failure time (days), acute nausea CR rate and adverse event incidence rate of this population. Therefore, palonosetron hydrochloride injection has the potential to be widely administered in China.

## Figures and Tables

**Table I:** Comparison of basic data (count data/grade data) between two groups.

Variable	Group A (%)	Group B (%)	Test	Statistic	P-value
Gender					
Male	31 (26.50)	32 (26.89)	Exact test		1.000
Female	86 (73.50)	87 (73.11)			
Total	117	119			
Central nervous system involvement					
No	113 (96.58)	117 (98.32)	Exact test		0.444
Yes	4 (3.42)	2 (1.68)			
Total	117	119			
Chemotherapy drugs					
Moderately emetogenic chemotherapy	22 (18.80)	21 (17.65)	Exact test		0.867
Severely emetogenic chemotherapy	95 (81.20)	98 (82.35)			
Total	117	119			
Chemotherapy times					
First	61 (52.14)	58 (48.74)	Exact test		0.606
Numerous times	56 (47.86)	61 (51.26)			
Total	117	119			
Other medical history					
No	87 (74.36)	86 (72.27)	Exact test		0.769
Yes	30 (25.64)	33 (27.73)			
Total	117	119			
Drug combination					
No	60 (51.28)	51 (42.86)	Exact test		0.240
Yes	57 (48.72)	68 (57.14)			
Total	117	119			
Tumor diagnosis					
Non-small cell lung cancer	27 (23.08)	23 (19.33)	CMH test	4.64	0.703
Colorectal cancer	4 (3.42)	4 (3.36)			
Gastric cancer	0 (0.00)	1 (0.84)			
Breast cancer	71 (60.68)	73 (61.34)			
Esophageal cancer	0 (0.00)	2 (1.68)			
Head and neck cancer	2 (1.71)	1 (0.84)			
Ovarian cancer	1 (0.85)	3 (2.52)			
Other	12 (10.26)	12 (10.08)			
Total	117	119			
Electrocardiogram					
Normal	72 (61.54)	67 (56.30)	CMH test	2.16	0.539
Abnormal, insignificance	33 (28.21)	43 (36.13)			
Abnormal, significance	8 (6.84)	5 (4.20)			
Unchecked	4 (3.42)	4 (3.36)			
Total	117	119			

CMH test, Cochran-Mantel-Haenszel test. Group A, experimental group; Group B, control group.

**Table II:** Comparison of basic data (count data) between two groups.

Variable	Group A	Group B	Test	Statistic	P-value
Age (years)					
N (missing)	117 (0)	119 (0)	Wilcoxon rank sum test	0.06	0.951
Mean ± SD	52.06±10.46	52.23±9.85			
Median (Q1–Q3)	53.00 (44.00–60.00)	53.00 (46.00–60.00)			
Min-Max	31.00–72.00	31.00–73.00			
Height (cm)					
N (missing)	117 (0)	119 (0)	Wilcoxon rank sum test	0.54	0.586
Mean ± SD	161.68±7.22	161.43±7.70			
Median (Q1–Q3)	160.00 (156.00–168.00)	160.00 (156.00–168.00)			
Min-Max	147.00–180.00	148.00–185.00			
Weight (kg)					
N (missing)	117 (0)	119 (0)	Group t-test	0.60	0.546
Mean ± SD	59.78±9.19	59.07±8.86			
Median (Q1–Q3)	59.00 (54.00–65.00)	58.50 (53.00–65.00)			
Min-Max	42.00–85.00	40.00–89.00			
Temperature (°C)					
N (missing)	117 (0)	119 (0)	Wilcoxon rank sum test	0.51	0.610
Mean ± SD	36.77±0.38	36.74±0.29			
Median (Q1–Q3)	36.80 (36.50–37.00)	36.80 (36.50–37.00)			
Min-Max	36.00–38.30	36.00–37.70			
Heart rate (bpm)					
N (missing)	117 (0)	119 (0)	Wilcoxon rank sum test	0.74	0.459
Mean ± SD	79.10±4.84	79.39±4.31			
Median (Q1–Q3)	80.00 (78.00–82.00)	80.00 (78.00–80.00)			
Min-Max	62.00–92.00	68.00–99.00			
Breath					
N (missing)	117 (0)	119 (0)	Wilcoxon rank sum test	0.17	0.864
Mean ± SD	19.03±1.12	19.08±1.18			
Median (Q1–Q3)	19.00 (18.00–20.00)	19.00 (18.00–20.00)			
Min-Max	15.00–21.00	17.00–22.00			
Systolic pressure (mmHg)					
N (missing)	117 (0)	119 (0)	Wilcoxon rank sum test	1.36	0.172
Mean ± SD	120.33±14.69	123.54±15.30			
Median (Q1–Q3)	120.00 (110.00–130.00)	120.00 (110.00–133.00)			
Min-Max	91.00–164.00	96.00–169.00			
Diastolic pressure (mmHg)					
N (missing)	117 (0)	119 (0)	Wilcoxon rank sum test	0.12	0.906
Mean ± SD	76.73±8.32	76.87±9.05			
Median (Q1–Q3)	78.00 (70.00–80.00)	77.00 (70.00–82.00)			
Min-Max	56.00–100.00	60.00–114.00			
KPS score					
N (missing)	117 (0)	119 (0)	Wilcoxon rank sum test	0.44	0.660
Mean ± SD	89.77±6.91	89.17±7.66			
Median (Q1–Q3)	90.00 (90.00–90.00)	90.00 (85.00–90.00)			
Min-Max	70.00–100.00	65.00–100.00			
Hemoglobin (g/l)					
N (missing)	117 (0)	119 (0)	Wilcoxon rank sum test	0.04	0.968
Mean ± SD	120.99±15.91	120.70±15.79			
Median (Q1–Q3)	121.00 (109.00–131.40)	122.00 (110.00–132.00)			
Min-Max	92.00–182.00	90.00–169.00			
White blood cell					
N (missing)	117 (0)	119 (0)	Wilcoxon rank sum test	0.12	0.907
Mean ± SD	6.65±2.83	6.58±2.69			
Median (Q1–Q3)	6.20 (5.00–7.40)	5.90 (5.10–7.40)			
Min-Max	3.00–22.40	2.70–24.20			
Neutrophil					
N (missing)	117 (0)	119 (0)	Wilcoxon rank sum test	0.12	0.903
Mean ± SD	4.50±2.52	4.47±2.57			
Median (Q1–Q3)	3.84 (3.00–5.20)	3.90 (2.90–5.30)			
Min-Max	1.80–18.80	1.50–21.80			
Platelet					
N (missing)	117 (0)	119 (0)	Wilcoxon rank sum test	0.52	0.603
Mean ± SD	251.25±91.56	248.16±93.08			
Median (Q1–Q3)	241.00 (183.00–303.00)	235.00 (180.00–298.00)			
Min-Max	71.00–741.00	88.00–565.00			
Total bilirubin (*μ*mmol/l)					
N (missing)	117 (0)	119 (0)	Wilcoxon rank sum test	1.78	0.075
Mean ± SD	9.04±4.32	10.33±4.92			
Median (Q1–Q3)	8.50 (6.50–12.00)	9.56 (6.80–13.20)			
Min-Max	0.16–24.30	0.26–24.20			
Direct bilirubin (*μ*mmol/l)					
N (missing)	117 (0)	119 (0)	Wilcoxon rank sum test	0.38	0.704
Mean ± SD	2.64±1.88	2.69±1.86			
Median (Q1–Q3)	2.30 (1.60–3.20)	2.40 (1.70–3.10)			
Min-Max	0.05–13.00	0.06–14.90			
ALT (U/l)					
N (missing)	117 (0)	119 (0)	Wilcoxon rank sum test	0.30	0.766
Mean ± SD	25.63±19.31	26.47±18.24			
Median (Q1–Q3)	20.00 (14.20–30.00)	21.00 (14.80–32.40)			
Min-Max	7.00–141.00	4.00–103.00			
AST (U/L)					
N (missing)	117 (0)	119 (0)	Wilcoxon rank sum test	0.96	0.335
Mean ± SD	24.86±13.97	24.42±12.99			
Median (Q1–Q3)	21.00 (17.40–28.80)	20.00 (17.00–27.00)			
Min-Max	11.00–122.00	11.00–77.00			
Albumin (g/l)					
N (missing)	117 (0)	119 (0)	Wilcoxon rank sum test	0.02	0.985
Mean ± SD	40.35±5.21	40.49±5.82			
Median (Q1–Q3)	40.50 (37.00–44.10)	40.30 (37.00–43.90)			
Min-Max	23.00–50.90	27.00–71.10			
Glucose (mmol/l)					
N (missing)	109 (8)	116 (3)	Wilcoxon rank sum test	1.57	0.115
Mean ± SD	12.06±24.42	8.65±16.03			
Median (Q1–Q3)	5.24 (4.80–5.82)	5.06 (4.73–5.76)			
Min-Max	4.00–139.00	3.90–88.00			
K (mmol/l)					
N (missing)	112 (5)	114 (5)	Wilcoxon rank sum test	0.47	0.642
Mean ± SD	4.16±0.40	4.13±0.50			
Median (Q1–Q3)	4.15 (3.96–4.41)	4.10 (3.87–4.46)			
Min-Max	3.10–5.35	1.44–5.66			
Na (mmol/l)					
N (missing)	112 (5)	114 (5)	Wilcoxon rank sum test	1.76	0.079
Mean ± SD	139.44±2.60	140.01±2.71			
Median (Q1–Q3)	139.95 (138.00–141.00)	140.15 (138.20–142.00)			
Min-Max	125.30–145.00	132.10–147.40			
Cl (mmol/l)					
N (missing)	112 (5)	114 (5)	Wilcoxon rank sum test	1.79	0.074
Mean ± SD	102.47±2.92	103.20±3.09			
Median (Q1–Q3)	102.90 (101.00–104.05)	103.25 (101.70–105.00)			
Min-Max	89.90–109.00	93.50–112.80			
BUN (mmol/l)					
N (missing)	117 (0)	119 (0)	Wilcoxon rank sum test	1.51	0.130
Mean ± SD	4.95±2.35	5.19±2.30			
Median (Q1–Q3)	4.33 (3.68–5.18)	4.63 (3.93–5.90)			
Min-Max	1.50–18.10	1.30–15.80			
Cr (*μ*mmol/l)					
N (missing)	117 (0)	119 (0)	Wilcoxon rank sum test	0.25	0.805
Mean ± SD	56.03±21.70	57.58±23.33			
Median (Q1–Q3)	56.30 (47.10–67.70)	54.90 (45.20–68.10)			
Min-Max	0.52–123.00	0.55–183.00			

Group A, experimental group; Group B, control group;. ALT, glutamic-pyruvic transaminase enzyme; AST, glutamic-oxalacetic transaminease; BUN, urea nitrogen.

**Table III:** Comparison of the complete remission rate of acute vomiting between the two groups.

Index	Experimental group (%)	Control group (%)	Statistics	P-value
0 grade	56 (49.12)	49 (42.24)		
1 grade	17 (14.91)	23 (19.83)		
2 grade	23 (20.18)	19 (16.38)		
3 grade	18 (15.79)	25 (21.55)		
Total	114	116		
Efficacy			0.89	0.346
Complete remission rate^a^	56 (49.12)	49 (42.24)	0.87	0.350

a Complete remission rate 95% confidence interval, -5.96-19.73.

**Table IV:** Comparison of the complete remission rate of delayed vomiting between the two groups.

Index	Experimental group (%)	Control group (%)	Statistics	P-value
Complete remission	59 (51.75)	36 (31.03)		
No complete remission	55 (48.25)	80 (68.97)		
Total	114	116		
Complete remission rate^a^	59 (51.75)	36 (31.03)	9.58	0.002

a Complete remission rate 95% confidence interval, 8.27–33.17.

**Table V:** Comparison of the complete remission rate of full vomiting between the two groups.

Index	Experimental group (%)	Control group (%)	Statistics	P-value
Complete remission	43 (37.72)	32 (27.59)		
No complete remission	71 (62.28)	84 (72.41)		
Total	114	116		
Complete remission rate^a^	43 (37.72)	32 (27.59)	2.40	0.121

a Complete remission rate 95% confidence interval, -1.92-22.19.

**Table VI:** Vomiting control time (hours) and comparison-FAS.

Index	Experimental group	Control group	Test	Statistic	P-value
N (missing)	73 (44)	85 (34)	Wilcoxon rank sum test	0.56	0.573
Mean ± SD	7.62±11.28	9.72±16.01			
Median (Q1–Q3)	3.00 (0.00–8.00)	3.70 (0.50–12.00)			
Min-Max	0.00–50.00	0.00–95.00			

FAS, full analysis set.

**Table VII:** Time to treatment failure (days) in the two groups and comparison-FAS.

Index	Experimental group	Control group	Test	Statistic	P-value
N (missing)	22 (95)	33 (86)	Wilcoxon rank sum test	0.37	0.712
Mean ± SD	1.41±1.01	1.36±0.70			
Median (Q1–Q3)	1.00 (1.00–1.00)	1.00 (1.00–1.00)			
Min-Max	1.00–5.00	1.00–3.00			

FAS, full analysis set.

**Table VIII:** Comparison of nausea complete response rate in the two groups; curative effect.

Index	Experimental group (%)	Control group (%)	Statistics	P-value
0 grade	37 (32.46)	32 (27.59)		
1 grade	49 (42.98)	44 (37.93)		
2 grade	25 (21.93)	35 (30.17)		
3 grade	3 (2.63)	5 (4.31)		
Total	114	116		
Efficacy			2.22	0.136

**Table IX:** Occurrence of adverse event in the two groups.

Events	Experimental group			Control group			P-value
	Times	Cases	Incidence (%)	Times	Cases	Incidence (%)	
Adverse events	119	68	57.63	145	81	67.50	0.141
Important adverse events	81	51	43.22	90	51	42.50	1.000
Serious adverse events	0	0	0.00	1	1	0.83	1.000
Adverse reaction	21	17	14.41	18	15	12.50	0.707
